# Unicameral bone cyst of the lunate in an adult: case report

**DOI:** 10.1186/1749-799X-5-79

**Published:** 2010-10-30

**Authors:** Hakan Gündeş, Mustafa Sahin, Tugrul Alici

**Affiliations:** 1 Maltepe University, School of Medicine, Department of orthopedics and hand surgery. Istanbul, Turkey

## Abstract

We report a case of a symptomatic unicameral (simple) bone cyst of the lunate in a 42-year- old woman. The lesion was treated with curettage and cancellous autogenous iliac bone grafting. At five years of follow-up the wrist was pain free, there were no limitations of motion, and the radiographs showed complete obliteration of the cavity. To the best of our knowledge, no other unicameral bone cyst of the lunate has been reported in an adult. Cysts with significant cavities at the carpal bones in an adult should be approached cautiously, as they may require early curettage and bone grafting for healing, before collapse and degenerative changes occur.

## Background

Unicameral bone cysts (UBCs), also known as simple bone cysts are benign, fluid-filled lesions involving the metaphysis of long bones [1-3]. On radiography they demonstrate a centrally located lytic lesion with well-defined margins [2]. The cyst wall is lined with a fibrous membrane which contains serous yellow fluid [2]. 80% of UBCs occur in the proximal humerus and proximal femur [1,3]. Most UBCs occur in childhood where one third of the cases will resolve spontaneously by skeletal maturity [1-3]. Occurrence of a single symptomatic radiolucent lesion in the lunate is rare [4,5]. A differential diagnosis of a painful radiolucent lesion in the lunate would include intraosseous ganglion, Kienböck's disease, osteoid osteoma, giant cell tumor, aneurysmal bone cyst and enchondroma [4-9]. The incidence of UBCs involving the wrist bones and lunate has not been clearly defined in the literature [10].

## Case presentation

**Figure 1:**
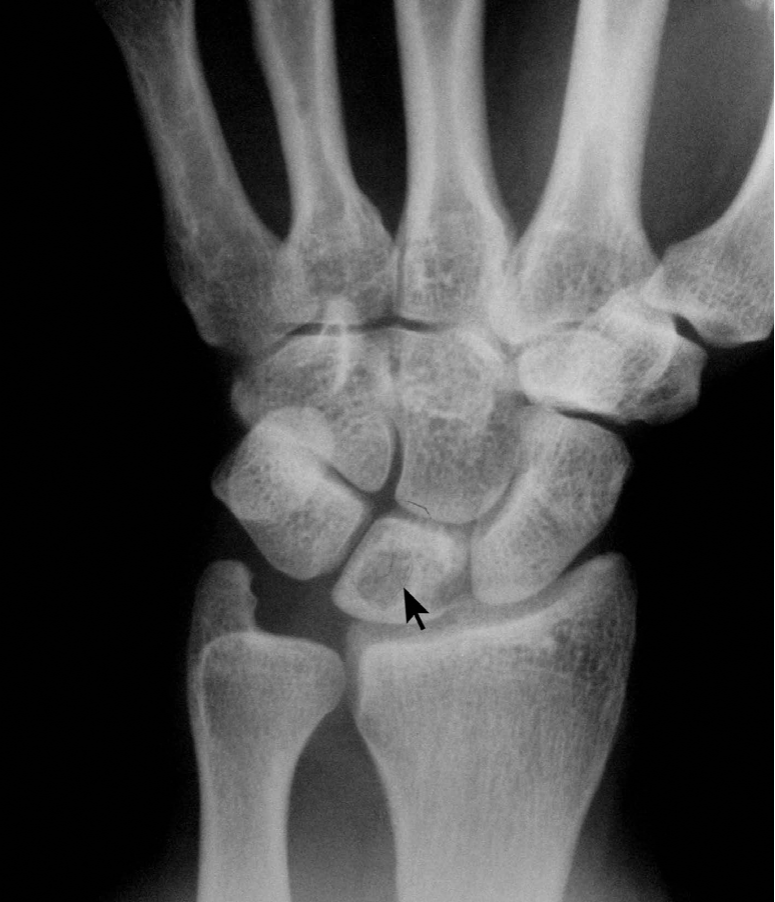
**PA radiograph of the right wrist**. There was a radiolucent lesion measuring 11 mm in diameter at the center of the lunate with round margins. There was no scalloping, septae formation or cortical thinning.

**Figure 2:**
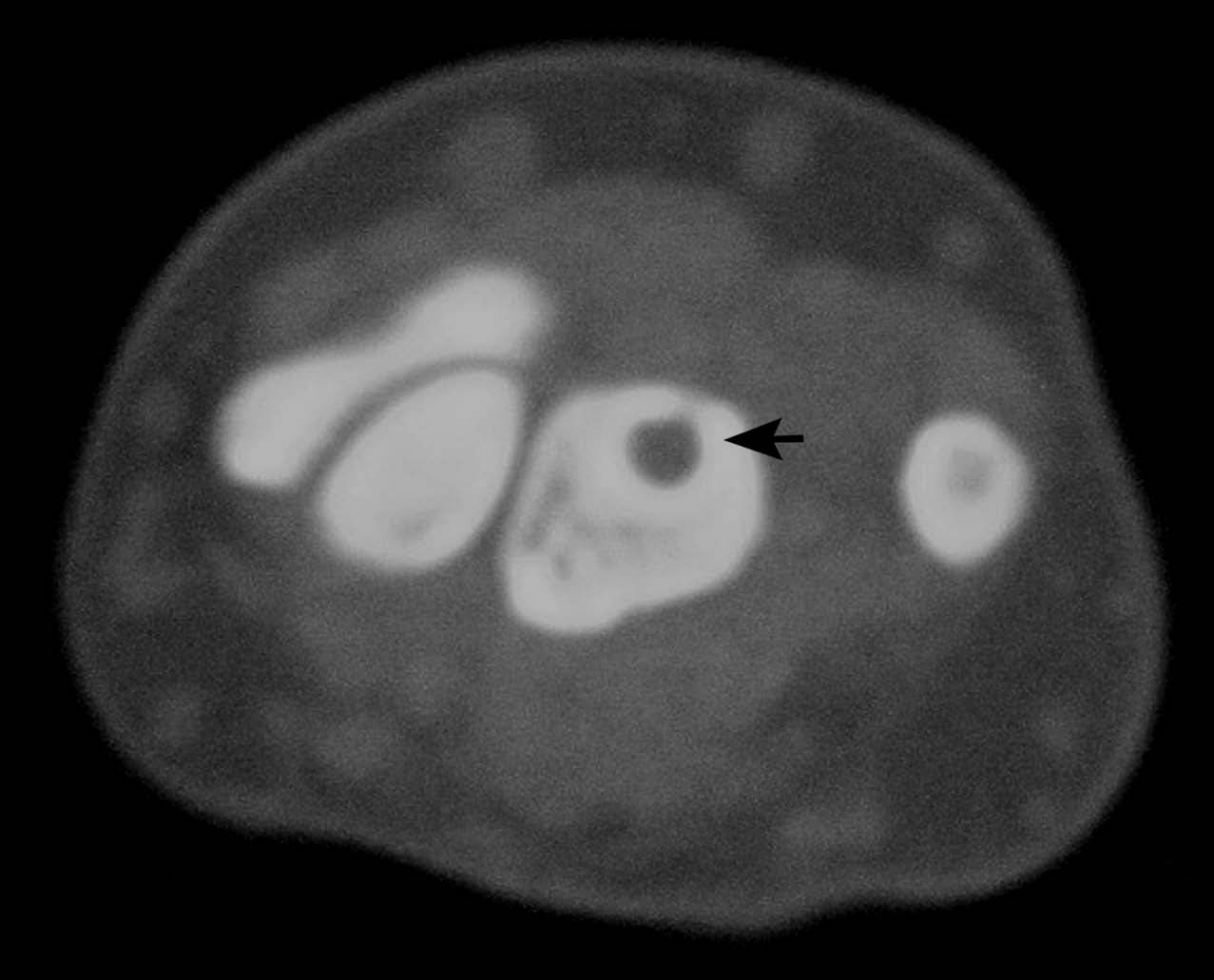
**Computed Tomography (CT) scans of the wrist revealed a round hypodense cystic lesion of 10 mm in diameter**.

**Figure 3:**
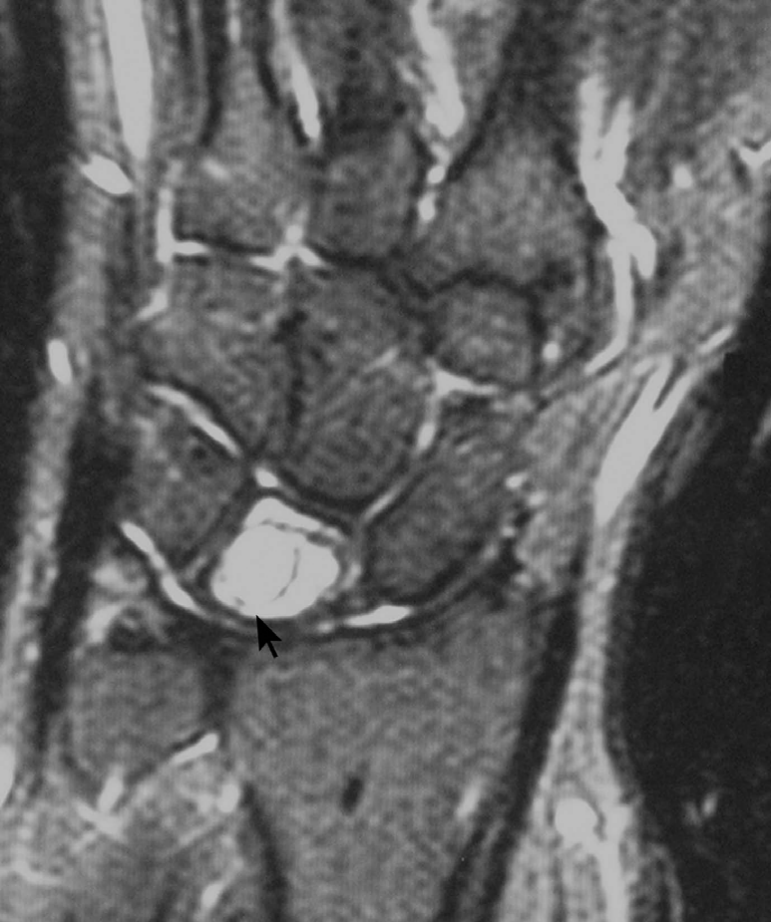
**Magnetic Resonance (MR) imaging views on fat-suppressed T2 weighted sequences have revealed a homogenous hyper-intense cystic lesion in the lunate with smooth and round contours**.

A 42-year-old woman was referred to the hand clinic with dull pain and discomfort in her right dominant wrist that had been present for six months. Pain was steady and not aggravated by use. Examination revealed very mild dorsal swelling of the wrist, with tenderness over lunate. The range of motion was slightly restricted in all directions. A specific limitation on wrist flexion and radial deviation was observed. A scaphoid shift test was negative. Routine biochemical tests, blood count and erythrocyte sedimentation rate (ESR) were within normal limits. AP and lateral radiograph of the wrist revealed a radiolucent lesion measuring 11 mm in diameter at the center of the lunate with round margins (Figure 1). There was no scalloping, septae formation or cortical thinning. Computed Tomography (CT) scans of the wrist revealed a round hypodense cystic lesion of 10 mm in diameter without septae formation (Figure 2). Magnetic Resonance (MR) imaging views on coronal fat-suppressed and axial and sagittal T2 weighted sequences have revealed a homogenous hyper-intense cystic lesion in the lunate with smooth and round contours (Figure 3).

**Figure 4:**
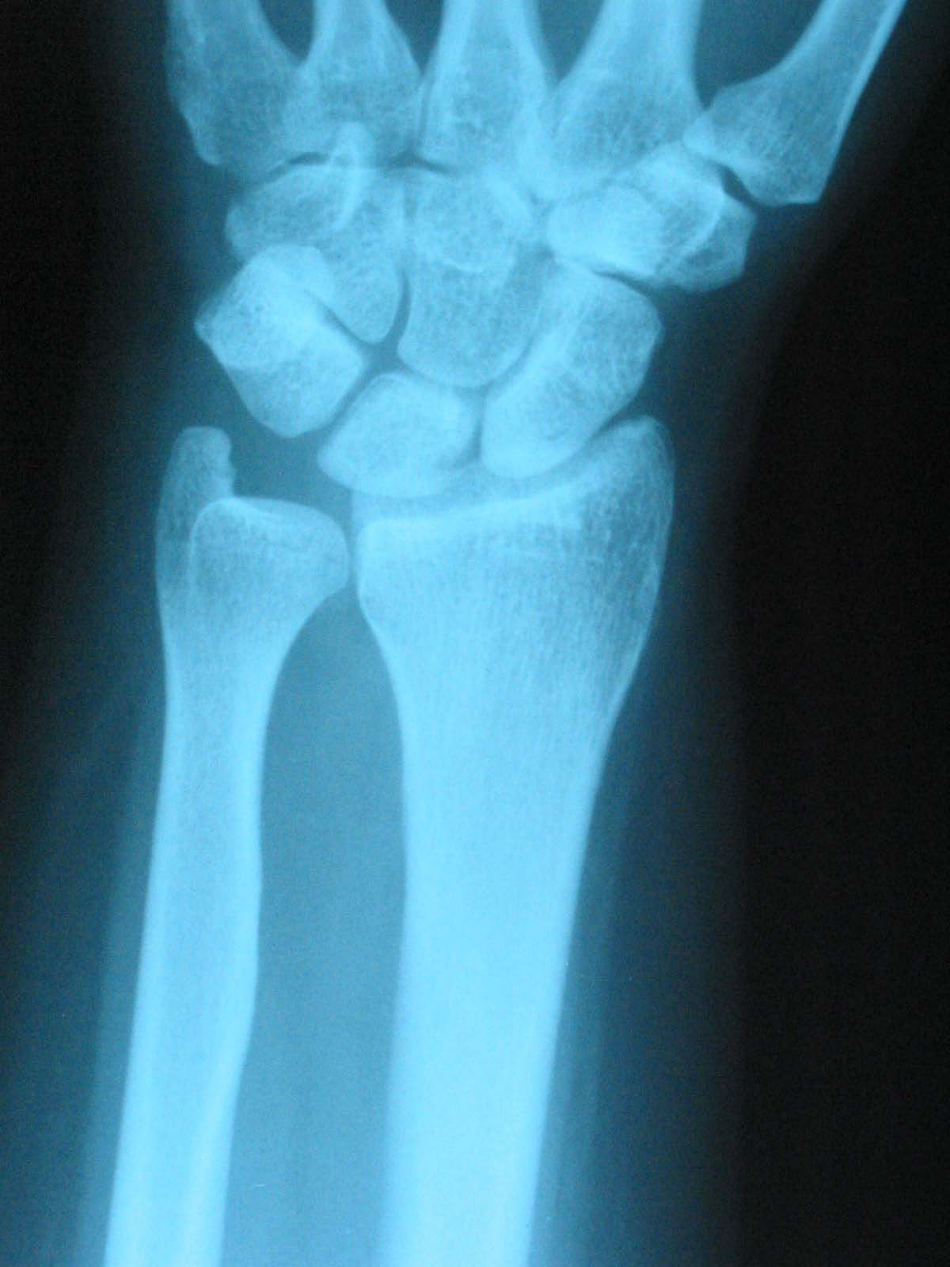
**A radiograph taken five years after the operation showed solid incorporation of the graft, and complete obliteration of the cavity**.

A dorsal longitudinal incision of 6 cm was made on the ulnar side of the Lister's tubercle, extending proximally and distally through the skin and subcutaneous tissue. The extensor retinaculum was sectioned between the third and fourth compartments, parallel to the incision. The third and fourth compartments were connected. Tendons were retracted and the capsule was exposed. The capsule was cut open through an H-shaped incision, allowing the evaluation of the proximal part of the capitate and the lunate fossa. Curettage was performed by opening a dorsal 3 mm cortical window through the cartilage. After the fluid was aspirated, the fibrous membrane-like tissue lining the cyst wall was curetted, and a power burr was not used. The cyst was packed with cancellous autogenous iliac bone chips. We preferred to utilize autogenous iliac bone over distal radius in order to increase the chance of incorporation [1]. The wrist was protected with a well padded splint for two weeks to alleviate the pain and discomfort. After that, active and passive range of motion exercises and strengthening had been instituted. The histopathological diagnosis was unicameral bone cyst. A radiograph that had been taken two years after the operation showed solid incorporation of the graft. At five years of follow-up, the wrist was pain free, there were no limitations of motion observed, and the radiograph showed complete obliteration of the cavity (Figure 4).

## Conclusions

Most diagnosed UBCs occur in childhood [1-3]. UBC etiology is unknown [1,3]. They account for 3% of all bone tumors, and usually involve the metaphysis of long bones, and have a predilection for the proximal humerus and proximal femur [2,3]. A debate exists whether treatment is necessary (because of spontaneous resolution) and what treatment is most appropriate [1]. Patients with UBCs usually present with a pathological fracture or a complaint of mild pain in the affected region [2,3]. The main indication for surgery is to prevent or treat pathological fracture [1]. Benign bone lesions are often treated with intralesional curettage, and autogenously bone grafts or various substitutes have been used to fill the defect [11]. Curettage alone is often the standard treatment for benign bone tumors giving the similar recurrence and fracture rates [11]. Described treatment options for a UBC include simple observation, curettage and grafting (autogenous or allogenous), steroids, demineralized bone matrix, and bone marrow injection [1,3]. The incidence of UBCs involving the wrist bones and lunate has not been clearly defined in the literature[10]. The differential diagnosis of a radiolucent lesion of the lunate most commonly includes an intraosseous ganglion cyst or osteoid osteoma [7]. Kienböck's disease, osteoid osteoma, giant cell tumor, enchondroma, aneurismal bone cyst (ABC), nonossifying fibroma and fibrous dysplasia are less likely possibilities [4-9]. There are no established guidelines for when and how to treat UBCs. Injections of steroids, demineralized bone matrix, and bone marrow aspirate have been reported as methods of treatment with various success rates [1,3]. Standard surgical treatment consists of curettage and cancellous bone grafting [3]. The main indication for surgical intervention is to prevent or treat a pathological fracture [1,3].

In our case herein, indications for surgery were clinical history of pain and radiographic findings of a cystic formation in the lunate.

UBCs of carpal bone in adulthood had been reported before [10]. This was a case report of bilateral unicameral bone cysts located in the hamate bones of a 22-year-old man [10]. Our patient was unique in that she had a UBC in her lunate bone. To the best of our knowledge, no other unicameral bone cyst of lunate has been reported in the literature. The etiology of this symptomatic lesion remains unknown. Cysts with such large cavities at the carpal bones in an adult should be approached cautiously. They may require early curettage and bone grafting for healing. Early treatment has its' definitive benefits as it prevents collapse and degenerative changes as in our case [8].

## Authors' contributions

HG carried out the operation, followed-up the patient and wrote the manuscript. MS and TA participated in writing and design of the manuscript. They also drafted the manuscript. All authors read and approved the final manuscript.

## Conflict of interest statement

Authors certifies that they have no commercial associations (e.g., consultancies, stock ownership, equity interest, patent/licensing arrangements, etc.) that might pose a conflict of interest in connection with the submitted article.

## Consent

Written informed consent was obtained from the patient for publication of this case report and accompanying images. A copy of the written consent is available for review by the Editor-in-Chief of this journal.
